# SVGenes: a library for rendering genomic features in scalable vector graphic format

**DOI:** 10.1093/bioinformatics/btt294

**Published:** 2013-06-06

**Authors:** Graham J. Etherington, Daniel MacLean

**Affiliations:** The Sainsbury Laboratory, Norwich Research Park, Norwich, NR4 7UH, UK

## Abstract

**Motivation:** Drawing genomic features in attractive and informative ways is a key task in visualization of genomics data. Scalable Vector Graphics (SVG) format is a modern and flexible open standard that provides advanced features including modular graphic design, advanced web interactivity and animation within a suitable client. SVGs do not suffer from loss of image quality on re-scaling and provide the ability to edit individual elements of a graphic on the whole object level independent of the whole image. These features make SVG a potentially useful format for the preparation of publication quality figures including genomic objects such as genes or sequencing coverage and for web applications that require rich user-interaction with the graphical elements.

**Results:** SVGenes is a Ruby-language library that uses SVG primitives to render typical genomic glyphs through a simple and flexible Ruby interface. The library implements a simple Page object that spaces and contains horizontal Track objects that in turn style, colour and positions features within them. Tracks are the level at which visual information is supplied providing the full styling capability of the SVG standard. Genomic entities like genes, transcripts and histograms are modelled in Glyph objects that are attached to a track and take advantage of SVG primitives to render the genomic features in a track as any of a selection of defined glyphs. The feature model within SVGenes is simple but flexible and not dependent on particular existing gene feature formats meaning graphics for any existing datasets can easily be created without need for conversion.

**Availability:** The library is provided as a Ruby Gem from https://rubygems.org/gems/bio-svgenes under the MIT license, and open source code is available at https://github.com/danmaclean/bioruby-svgenes also under the MIT License.

**Contact:** dan.maclean@tsl.ac.uk

## 1 INTRODUCTION

Visualization, analysis and communication of genome data is an important task in genomics. Numerous desktop computer programs exist for rendering images of genomic data, usually in analytic pipelines including Artemis (Carver *et al.*, 2008). Genome browsers such as Gbrowse (Stein *et al.*, 2002), JBrowse (Skinner *et al.*, 2009), Savant (Fiume *et al.*, 2010) and IGV (Thorvaldsdóttir *et al.*, 2013) provide interactive visualization of the data for whole genomes and draft assemblies. Output from these is typically limited to an exported bitmap or screen grab in the program’s particular fixed style. Graphics libraries such as GD and ImageMagick have been used in projects like BioPerl (Stajich *et al.*, 2002) and BioRuby (Goto *et al.*, 2010) to create uniquely styled bitmap images like PNG and JPEG programmatically. BioRubys bio-graphics package has similar functionality to bio-svgenes and relies on external libraries such as Cairo, Pango and ImageMagick. The Bio.Graphics module in Biopython (Cock *et al.*, 2009) also supports output in SVG through the use of third-party software [ReportLab (http://www.reportlab.com/)]. Bitmap images are limited in that they are not easy to re-annotate, re-scale and often cannot be reproduced for publication or presentation with high-fidelity because of limitations of the original bitmaps. Bitmaps can be difficult to manipulate and are not easily amenable to the addition of interactive features. Interactive graphics can be provided in web-browsers through JavaScript libraries such as D3.js but there are no such libraries available specifically for easy rendering of genomic data. Scalable Vector Graphics (SVG) is an XML-based graphic standard under development by the World Wide Web Consortium that provides many advantages for those seeking to produce rich, attractive images. SVG is a vector format so does not suffer image quality degradation on rescaling, has advanced image features such as alpha masks and filter effects, web-interactivity and can be styled with Cascading Style Sheets. Furthermore, as a text-based format, SVG is well suited for searching and indexing in databases and is amenable to lossless compression. SVG can be rendered by modern web-browsers and graphics software including Adobe’s Illustrator and the open source Inkscape programs. SVG output is available from some applications. CGView (Grant *et al.*, 2012) and Circos (Krzywinski *et al.*, 2009) are good tools for viewing circular genomes in particular. GenomeDiagram (Pritchard *et al.*, 2006) is designed to display large amounts of comparative genomics data. MGV (Kerkhoven *et al.*, 2004) is a database-driven web application designed specifically for microbial data and AnnotationSketch (Steinbiss *et al.*, 2009), which is dependant on third-party software. SVGenes is a pure Ruby language library that allows a user to set styles for tracks of genomic features and will automatically layout and generate SVG images composed of several pre-defined genomics glyphs, including genes, transcripts, data and single-nucleotide features.

## 2 APPROACH

SVGenes uses a simple feature Page-Track-Feature model to organize the genomic data and to apply style information provided to it.

### 2.1 The page and track object

The page object represents the area into which feature tracks are drawn, it has straightforward width, height and background attributes. Height is not fixed and is recalculated if more space is required to render all the constituent features at the specified sizes. The background attribute of the page can be styled, and an automatically generated scale bar is created and added to the top of the rendered page. Instantiating new track objects is the main way that styling information is specified, the track attributes set the final visual style of the genomic features and is responsible for placing them within the track on the page.

### 2.2 Glyphs and feature objects

**Fig. 1.**
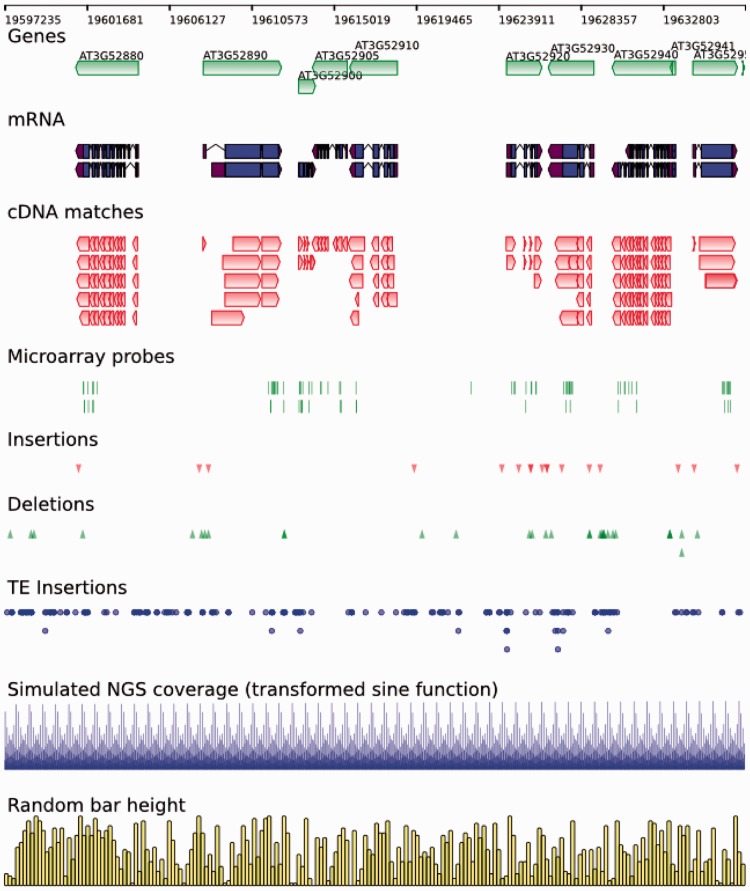
Rendering of features from the TAIR 10 annotation of the *Arabidopsis* genome (Lamesch *et al.*, 2012). The region shown is Chromosome 3: 19 597 235–19 637 249. Tracks contain (from top to bottom) (i) Genes with the ’directed’ glyph, (ii) mRNAs with the ‘transcript’ glyph, (iii) cDNA matches with the ‘directed’ glyph, (iv) microarray probes with the ‘generic’ glyph, (v) insertions with the ‘down triangle’ glyph, (vi) deletions with the ‘up triangle’ glyph, (vii) TE insertions with the ‘circle’ glyph, (viii) a data track showing simulated NGS data (height calculated from a sine function of the genome position) and (ix) a data track showing random bar heights

SVGenes can render genomic features using various glyphs including gene, transcript and point features. Data tracks representing, e.g. sequence read coverage can be rendered as histograms and the flexible styling options allow for a great deal of variety of appearance (Fig. 1 shows some examples). Each glyph takes style information from the track, and full SVG styling syntax can be used for arbitrary styling information including opacity settings. HTML colours and some pre-defined gradient fills are available through keyword declaration, greatly simplifying basic styling. The feature object represents genomic features simply and flexibly. As a minimum, start and stop positions are required for the basic glyphs. Grouped features such as transcripts are represented by start and stop information for the parent object and start and stops for the block elements within, such as exons and untranslated regions. Data glyphs are bars with start, width and height elements.

### 2.3 Workflow

SVGenes provides programmatic and configuration-based rendering workflows. Within a Ruby script, a user may manually instantiate a page object and attach tracks as required, then create and add the feature objects to the appropriate tracks. This workflow does not rely on any particular feature file format. For input from the popular GFF format, a configuration-based workflow is provided. In this, the user is able to create a JSON configuration file that describes each track and contains links to a file containing the features or data values to be rendered in each track.

## 3 CONCLUSION

SVGenes is a useful and flexible library for creating easily manipulated, high-quality, web-friendly images of genomic data quickly and easily in SVG format without embedding a bitmap. The library can be used for visualization at many levels; in high-throughput pipelines and web applications, but individual users preparing figures for publication will also find the library extremely useful, as the individual elements of the images can be independently manipulated and annotated and composited.
